# Immunoglobulin-G4 laryngitis with co-existing Peyronie’s disease

**DOI:** 10.1016/j.bjorl.2024.101395

**Published:** 2024-01-25

**Authors:** Shivanchan Rajmohan, Chuanyu Gao, Kajaanan Rajmohan, Shivun Khosla, Lisa Pitkin

**Affiliations:** a Frimley Park Hospital, Frimley Health NHS Foundation Trust, Camberley, United Kingdom; b London Northwest Healthcare NHS Trust, United Kingdom

## Introduction

Immunoglobulin-G4 (IgG4) related disease whilst systemic has rarely made an appearance in the larynx. This is the first reported case of IgG4 autoimmune laryngitis coexisting with Peyronie’s Disease (PD) within the same subject.

## Case report

A 29-year-old male groundsman presented to the haematology outpatient department with 3 months of fluctuating cervical lymphadenopathy and 1–2 pounds of weight loss. Haematologists referred him to Otolaryngology in January 2021 for globus sensation within the throat associated with altered swallowing, 8-months history of a “croaky” voice, and postnasal space congestion. He denied fever, referred otalgia, odynophagia, night sweats and neck pain.

Past medical history comprised of hypertension controlled with propranolol and anxiety managed with citalopram. There was no history of previous radiotherapy, trauma, or surgeries. He was a non-smoker and did not drink alcohol. Family history was positive for multiple myeloma in his uncle.

**Figure 1:**
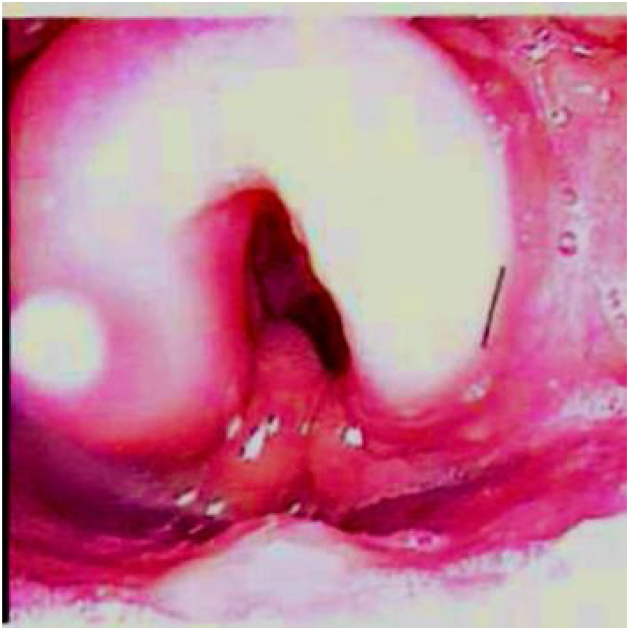
The first flexible nasoendoscopic view of the larynx demonstrating supraglottic oedema.

Comprehensive head and neck examination revealed no palpable lymphadenopathy with an unremarkable oral cavity and oropharynx. Flexible nasoendoscopy noted symmetrical oedema of the supraglottic larynx (Fig. 1) not typical of acute supraglottitis or any sinister pathology. Cardiorespiratory and abdominal examinations were normal.

His blood parameters (Table 1) collectively insinuated an underlying neutrophilic inflammatory process. Radiologically, his chest radiograph was unremarkable and his neck ultrasound demonstrated morphologically normal lymph nodes with the largest being 1.5 × 1.1 cm. Repeat neck ultrasound one month later illustrated normal sized lymph nodes. Abdominal ultrasound was unremarkable with no evidence of organomegaly.

**Table 1:** Table displaying blood parameters.

White cell count	11.5 × 10^9^/L (4.5–11)
Neutrophil	9.7 × 10^9^/L (1.5–8)
Lymphocytes	1.1 × 10^9^/L (1–4)
Haemoglobin	143 g/L (130–180)
Erythrocyte sedimentation rate (ESR)	85 mm/h (0–15)
Platelet	361 × 10^9^/L (150–450)
Creatinine	76 umoL/L (64–104)
C-reactive protein (CRP)	45 mg/L (0–9.9)
Lactate dehydrogenase	Normal
Direct Antiglobulin test (DAT)	Negative
Cytomegalovirus viral load	Undetected
Epstein Barr virus viral load	Undetected
Autoimmune screen (Rheumatoid factor, Anti-nuclear antibody, Myeloperoxidase antibody, Proteinase 3 antibody, Anti-mitochondrial antibody, Gastric parietal cell antibody, Liver kidney microsomal antibody and Smooth muscle antibody)	Negative
IgG1	6.21 g/L (3.2–10.2)
IgG2	8.14 g/L (1.2–6.6)
IgG3	0.42 g/L (0.2–1.9)
IgG4	1.91 g/L (0–1.3)
IgG1	6.21 g/L (3.2–10.2)

He was started on 2-weeks of oral co-amoxiclav and a tapering course of oral prednisolone (starting at 30 mg) with omeprazole cover. His symptoms resolved following oral steroids and antibiotics however on terminating the steroids he noticed a relapse of his altered voice and abnormal globus sensation in his throat.

**Figure 2:**
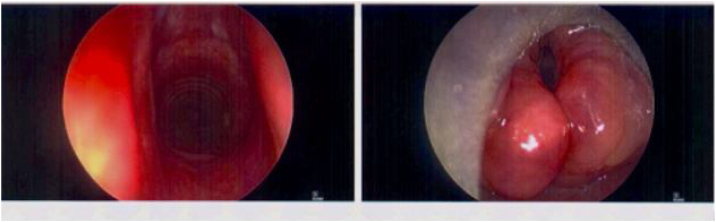
Intra-operative microlaryngoscopic image of larynx illustrating diffuse bilateral arytenoid swelling.

Repeat nasoendoscopy confirmed diffuse symmetrical supraglottic swelling; hence a general anaesthetic biopsy under Transnasal Humidified Rapid-Insufflation Ventilatory Exchange (THRIVE) was scheduled prior to recommencing a 3-week oral prednisolone tapering course (starting at 30 mg). Right arytenoid biopsy was obtained via suspension microlaryngoscopy in April 2021(Fig. 2).

Histology of the right arytenoid demonstrated granulomatous inflammation with initial differential diagnoses including sarcoidosis and IgG4 disease. The stroma showed a moderately dense lymphoplasmacytic inflammatory infiltrate including lymphoid follicle formation. Immunohistochemistry exposed innumerable IgG + plasma cells with IgG4 + cell number >50 per hpf and polytypic light chains. A few sarcoid type non-caseating microgranulomata were also visible. He had elevated immunoglobulins and lights chains without monoclonality. A definitive diagnosis of IgG4 autoimmune laryngitis was declared with elevated IgG4 and IgG2 titres.

The patient was reviewed by the rheumatology team and started on weekly 15 mg methotrexate and 10 mg folic acid in April 2021. Blood tests occurred monthly for the first 3-months followed by 3-monthly blood tests.

**Figure 3:**
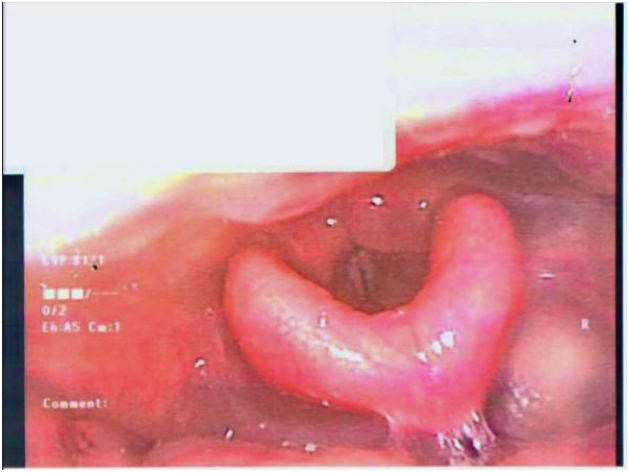
Repeat flexible nasoendoscopy 1 month after starting methotrexate. Note the improved laryngeal inlet appearance.

1-month follow-up post-immunosuppression therapy noted reduction in reported symptoms with objective improvement in laryngeal oedema on nasoendoscopy (Fig. 3).

After 5-months of methotrexate therapy, there was an 80% improvement in overall oedema however marked oedema of the left arytenoid complex persisted. Thus, the methotrexate dose was increased to 20 mg weekly. The provisional plan was to consider subcutaneous methotrexate if no improvement was seen with oral methotrexate.

Subsequent follow up in June 2022 (14-months post methotrexate initiation) demonstrated a normal larynx resulting in the decision to reduce his methotrexate to 15 mg weekly for a month and then 10 mg weekly thereafter. Further review at 19-months and 24-months post methotrexate initiation confirmed no recurrence.

Coincidentally, the patient was referred to the urology team in July 2018 for 45 degree acquired penile curvature present on erection with a palpable plaque on the dorsal aspect of his distal penis. Patient was diagnosed with PD and was prescribed vitamin E. At his 3-year follow up in September 2021, the penile dorsal curvature significantly improved measuring at 20–30 degrees with no indication for surgical correction.

## Discussion

To our knowledge is the first case describing IgG4 autoimmune laryngitis in a patient with PD. The administration of systemic steroids and methotrexate significantly improved his laryngeal oedema leading to remission but notably also positively impacted his PD.

Symptomatology described in laryngeal IgG4 disease include breathlessness, dysphagia, cough, voice change and stridor^1^ conforming with the symptoms faced by our patient.

While surgical correction remains the conventional approach for severe PD, urologists have explored alternative oral therapies. This includes Vitamin E, colchicine, tamoxifen, carnitine, phosphodiesterase-5 inhibitors, potassium para-aminobenzoate, pentoxifylline, and omega-3 fatty acids.^2^ Notably, Vitamin E supplementation operates at a molecular level, reducing oxidative stress and promoting tissue healing, although there is mixed evidence of its role in PD.^2^

Interestingly, there have been rare instances of PD emerging following initiation of methotrexate for inflammatory arthropathies and psoriatic dermatitis, resulting in discontinuation of methotrexate.^3^ However, in our case the contrary outcome was observed with improvement in penile deformity associated with PD six months after starting methotrexate. Chen et al.^4^ similarly documented the positive effect on PD when methotrexate was used to treat scleroderma. This improvement in PD is possibly related to the significantly elevated IgG4 titres noted in certain PD cohorts.^5^ Therefore one may postulate that in systemic rheumatological conditions with associated PD, addressing the underlying systemic condition may benefit concurrent PD and should be first line management for PD prior to exploring surgical avenues.^4^

Those with suspected IgG4 laryngeal disease should have prompt biopsies to exclude other sinister pathologies. Our case uniquely established simultaneously elevated IgG4 and IgG2 titres; further research is warranted on relevance of raised IgG2 levels in IgG4 disease and whether it has implications on severity of IgG4-related disease, choice, and effectiveness of treatments.

Moreover, our patient also incorporated Vitamin E into their PD treatment regimen, which could potentially complement the effects of methotrexate in individuals with systemic rheumatological conditions. However, further research is required to explore this potential synergy.^2^

## Conclusion

This is the first case report outlining biopsy proven IgG4 laryngitis in a patient with Peyronie’s disease with satisfactory improvement in both disease entities following methotrexate, oral steroid therapy and vitamin E.

## Funding

The authors did not receive any specific grant from funding agencies in the public, commercial, or not-for-profit sectors.

## Ethical approval

Informed consent was obtained from patient to publish this case report and images. All procedures performed abide ethical standards of Helsinki Declaration (as revised in 2013).

## Conflicts of interest

The authors declare no conflicts of interest.
